# Recombinant human leptin treatment in genetic lipodystrophic syndromes: the long-term Spanish experience

**DOI:** 10.1007/s12020-014-0450-4

**Published:** 2014-11-04

**Authors:** David Araujo-Vilar, Sofía Sánchez-Iglesias, Cristina Guillín-Amarelle, Ana Castro, Mary Lage, Marcos Pazos, José Manuel Rial, Javier Blasco, Encarna Guillén-Navarro, Rosario Domingo-Jiménez, María Ruiz del Campo, Blanca González-Méndez, Felipe F. Casanueva

**Affiliations:** 1 Division of Endocrinology and Nutrition, University Clinical Hospital of Santiago de Compostela, Santiago de Compostela, Spain; 2 UETeM-Molecular Pathology Group, Department of Medicine, IDIS-CIMUS-Facultade de Medicina, University of Santiago de Compostela, Avda de Barcelona s/n, 15707 Santiago de Compostela, Spain; 3 Division of Paediatrics, Hospital Nª Sª Candelaria, Tenerife, Canary Islands Spain; 4 Division of Paediatrics, Hospital Regional Universitario Carlos Haya, Malaga, Spain; 5 Division of Medical Genetics, Department of Paediatrics, University Clínical Hospital “Virgen de la Arrixaca”, Murcia, Spain; 6 Department of Medical Genetics, UCAM-Catholic University of Murcia, Murcia, Spain; 7 Centro de Investigación Biomédica en Red de Enfermedades Raras (CIBERER), Instituto de Salud Carlos III (ISCIII), Madrid, Spain; 8 Paediatric Neurology, Department of Paediatrics, University Clínical Hospital “Virgen de la Arrixaca”, Murcia, Spain; 9 Division of Paediatrics, Hospital San Pedro, Logroño, Spain

**Keywords:** Genetic lipodystrophy, Berardinelli-Seip syndrome, Familial partial lipodystrophy, Human recombinant leptin, Insulin resistance, Hypertriglyceridemia, Hepatic steatosis

## Abstract

Lipodystrophies are a group of diseases mainly characterized by a loss of adipose tissue and frequently associated with insulin resistance, hypertriglyceridemia, and hepatic steatosis. In uncommon lipodystrophies, these complications frequently are difficult to control with conventional therapeutic approaches. This retrospective study addressed the effectiveness of recombinant methionyl leptin (metreleptin) for improving glucose metabolism, lipid profile, and hepatic steatosis in patients with genetic lipodystrophic syndromes. We studied nine patients (five females and four males) with genetic lipodystrophies [seven with Berardinelli-Seip syndrome, one with atypical progeroid syndrome, and one with type 2 familial partial lipodystrophy (FPLD)]. Six patients were children under age 9 years, and all patients had baseline triglycerides levels >2.26 mmol/L and hepatic steatosis; six had poorly controlled diabetes mellitus. Metreleptin was self-administered subcutaneously daily at a final dose that ranged between 0.05 and 0.24 mg/(kg day) [median: 0.08 mg/(kg day)] according to the body weight. The duration of treatment ranged from 9 months to 5 years, 9 months (median: 3 years). Plasma glucose, hemoglobin A1c (Hb A1c), lipid profile, plasma insulin and leptin, and hepatic enzymes were evaluated at baseline and at least every 6 months. Except for the patient with FPLD, metreleptin replacement significantly improved metabolic control (Hb A1c: from 10.4 to 7.1 %, *p* < 0.05). Plasma triglycerides were reduced 76 % on average, and hepatic enzymes decreased more than 65 %. This study extends knowledge about metreleptin replacement in genetic lipodystrophies, bearing out its effectiveness for long periods of time.

## Introduction

Lipodystrophies are a group of diseases mainly characterized by a loss or lack of adipose tissue, although in some cases, some areas of lipohypertrophy also appear [1]. Frequently, lipodystrophic syndromes are associated with metabolic and hepatic disturbances, such as insulin resistance, atherogenic dyslipidaemia, and hepatic steatosis. These complications are usually responsible for serious co-morbidities (diabetes mellitus, cardiovascular diseases, acute pancreatitis, and cirrhosis) and mortality. As fat loss becomes more severe, associated complications will become more severe.

Lipodystrophies are classified into acquired and genetically determined forms, and excluding HIV-associated lipodystrophy, the other types are extremely uncommon [1]. No cure for lipodystrophies exists, and treatment targets controlling complications by standard therapeutical approaches, and, in some cases, applying surgical correction of lipohypo- and/or lipohypertrophic affected body areas [2].

Since 2002 [3], recombinant human methionyl leptin (metreleptin, Amylin Pharmaceuticals, San Diego, CA, USA) has been employed to treat the metabolic and hepatic complications of rare lipodystrophies, with reasonable results in terms of diabetes control, reduced hypertriglyceridemia, and improvement of hepatic steatosis [4]. This treatment seems to be effective for long periods [5] and is well tolerated with few side effects.

Although metreleptin was approved by the Japanese Health Authorities in 2013 and by the US Food and Drug Administration more recently [www.fda.gov/newsevents/newsroom/pressannouncements/ucm387060.htm] only for rare lipodystrophic syndromes, some limitations [6] exist in relation to the open-label character of these studies, obviously associated with the infrequent nature of these syndromes. In keeping with the goal of obtaining more evidence of the effectiveness of human recombinant leptin in treating uncommon lipodystrophies, we present our experience of using this hormone for nine patients with different rare lipodystrophic syndromes. The aim of this work was to confirm the efficacy of metreleptin for improving metabolic control, hypertriglyceridemia, and hepatic steatosis in patients with genetic lipodystrophies.

## Patients and methods

The Agencia Española del Medicamento approved the treatment with metreleptin for these patients as compassionate use, and the study was conducted according to the ethical guidelines of the Helsinki Declaration. Patients or their parents gave informed consent for participation in the study and publication of clinical and genetic information.

### Patients and study design

Nine patients with genetic lipodystrophic syndromes were enrolled. All of the patients except one [with familial partial lipodystrophy (FPLD)] had generalized lipodystrophy: seven with congenital generalized lipodystrophy (Berardinelli-Seip Syndrome, BS) and one with atypical progeroid syndrome (APS). The genetic, demographic, and clinical baseline features of these patients are shown in Table 1.

**Table 1:** Genetic and general features of the lipodystrophic patients before metreleptin treatment

Patient #	Origin	Lipodystrophy type	Gene	Mutations	Sex	Age	Duration of disease	Fat lack	DM	HBP	HyperTG
1	Spain	Berardinelli-Seip	BSCL2	c. 517dupA	M	22 years	22 years	G	Yes	Yes	Yes
2	Morocco	Berardinelli-Seip	Unknown^a^	Unknown	F	23 months	23 months	G	No	No	Yes
3	Spain	Berardinelli-Seip	BSCL2	c.985C>T/c.507_511del	F	37 months	37 months	G	No	No	Yes
4	Spain	Berardinelli-Seip	BSCL2	c.385_386delinsGGA/c.517dupA	F	21 years	21 years	G	Yes	No	Yes
5	Spain	Berardinelli-Seip	BSCL2	c.385_386delinsGGA/c.517dupA	M	8 years	8 years	G	Yes	No	Yes
6	Spain	Berardinelli-Seip	BSCL2	c.385_386delinsGGA/c.517dupA	M	8 years	8 years	G	No^b^	No	Yes
7	Pakistan	Berardinelli-Seip	AGPAT2	c.755_763 del TGAGGACCA	F	8.8 years	8.8 years	G	Yes	No	Yes
8	Spain	Atypical progeroid syndrome	LMNA	c.29C>T	M	8 years	2 years	G	Yes	No	Yes
9	Spain	FPLD 2	LMNA	c.895 A>G	F	43 years	31 years	P	Yes	Yes	Yes

Patient #	Acanthosis	Pancreatitis	Hepatic steatosis	Cardiomyopathy	Intellectual disability	Diabetic complications	Treatment
1	Yes	No	Yes	No	Mild	Nephropathy	Metformin/pioglitazone (30 mg)/insulin (2.2 UI/kg) Fenofibrate/n-3 FFA Enalapril/losartan Amlodipine
2	Yes	No	Yes	No	No^c^	NA	Animal fat-free diet
3	Yes	No	Yes	Hypertrophic cardiomyopathy	Mild^d^	NA	Animal fat-free diet
4	No	Yes	Yes	Hypertrophic cardiomyopathy Aortic and pulmonary stenosis	Mild	Proliferative retinopathy/nephropathy/peripheral arterial disease/polyneuropathy	Metformin/pioglitazone/insulin (3.9 IU/kg)/fenofibrate/clopidogrel/pentoxifylline
5	Yes	Yes	Yes	Hypertrophic cardiomyopathy Aortic stenosis	Mild	None	Metformin
6	Yes	No	Yes	Hypertrophic cardiomyopathy Aortic stenosis	Mild	NA	Metformin
7	Yes	No	Yes	Hypertrophic cardiomyopathy Aortic stenosis	No	None	Metformin/insulin (3.2 UI/kg)
8	No^e^	No	Yes	Dilated cardiomyopathy	No	None	Metformin Aspirin/digoxin/furosemide Captopril/bisoprolol
9	Yes	No	Yes	No	No	None	Pioglitazone/Insulin (1.4 UI/kg) Fenofibrate/FFA n-3 Atorvastatin/ezetimibe Valsartan/hydrochlorothiazide/amlodipine

*DM* diabetes mellitus, *HyperTG* hypertriglyceridemia, *HBP* high blood pressure, *G* generalized, *P* partial, *NA* not applicable, *FFA* free fatty acid

^a^No mutations in AGPAT2, BSCL2, or CAV1 genes

^b^Impaired glucose tolerance

^c^Hyperactivity

^d^Psychomotor delay

^e^Leukomelanodermic papulas

The inclusion criteria were the presence of a genetic lipodystrophic syndrome plus diabetes mellitus, defined according to the criteria of the American Diabetes Association [7], and/or plasma triglycerides higher than 2.26 mmol/L (200 mg/dL) and/or being on triglycerides-lowering drugs. Exclusion criteria were pregnancy, serious liver disease, cancer, or renal failure. Patient ages ranged from 23 months to 44 years, and five patients were male and four female. The study was designed as a retrospective, open-label study at the Complexo Hospitalario Universitario de Santiago de Compostela (Spain). Metreleptin was kindly provided first by Amylin Pharmaceuticals (San Diego, CA, USA) and later by AstraZeneca (London, UK), although all of the data were held by the academic investigators. No placebo-treated control group was included because of the rarity and severity of these syndromes.

Metreleptin was self-administered (or parent-administered) subcutaneously every 12 or 24 h, depending on the supplied volume (every 12 h in those receiving more than 1 mL (5.14 mg/d), patients #1 and #9). The replacement dose was calculated based on weight, and the final dose ranged between 0.05 and 0.24 mg/kg/day, with a median of 0.08 mg/kg/day, and was adjusted to achieve metabolic control, taking weight loss into account [4, 5]. Patients were seen every month for the first 6 months, and every 3 months for the rest of the first year, and then every 6–12 months thereafter. For patients on insulin treatment, the dose was reduced (20 %) every 3 months if the hemoglobin A1c (Hb A1c) value fell below 7 %. Other diabetes medications were stopped or the dose reduced if a patient reached good metabolic control. Lipid-lowering medication was stopped when plasma triglycerides were under 2.26 mmol/L (200 mg/dL). The possible side effects were self- or parent reported in every visit.

### Methods

Height and body weight were measured using a stadiometer and a digital balance. The waist circumference was taken using a flexible tape as the smallest standing horizontal circumference between the ribs and the iliac crest.

Fasting serum samples were analyzed for glucose, triglycerides, high-density lipoprotein-cholesterol (HDL-c), leptin and insulin, as described previously [8]. Blood Hb A1c was measured using ion-exchange high-performance liquid chromatography (Bio-Rad Laboratories Inc., Hercules, CA, USA). Alanine transaminase (ALT), aspartate transaminase (AST), and gamma-glutamyltransferase were determined by enzymatic methods using an ADVIA analyzer (Siemens, Bayer Diagnostics, Tarrytown, NY, USA). Thyroid-stimulating hormone, free thyroxine, and free triiodothyronine were measured by chemiluminescence using ADVIA Centaur (Bayer Diagnostics, Tarrytown, NY, USA).

### Statistical analysis

Data are shown as the mean ± standard deviation. Because of the small number of patients and the non-normal distribution of the variables, non-parametric analysis was carried out using the Wilcoxon signed-rank test. A *p* value of less than 0.05 was taken to indicate statistical significance. All analyses were carried out using the IBM SPSS 22.0 package.

## Results

**Fig. 1:**
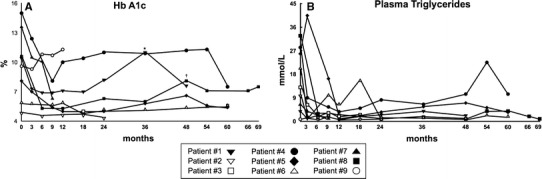
Time-course of Hb A1c (**a**) and plasma triglycerides (**b**) during metreleptin treatment. *1 year without metreleptin (patient #1); ^†^heart transplant (patient #8)

Anthropometric and auxological data are shown in Table 2. Metreleptin treatment was well tolerated for long periods of time (in some cases more than 5 years) without remarkable side effects. Treatment duration ranged from 9 months to 5 years, 9 months (median: 3 years). Only one patient (#9) reported transitory nauseas at the beginning of treatment (first week). Patient #1 voluntarily stopped metreleptin after 2 years because of the appearance of proximal lower limb myopathy, which was not considered related to the drug. The muscular symptoms spontaneously disappeared 6 months later, and metreleptin was resumed after one year because of a serious worsening of metabolic control (Fig. 1a).

**Table 2:** Anthropometric and auxological data for the lipodystrophic patients before and after metreleptin treatment

Patient #	Age	Months of treatment	Metreleptin dose (mg/kg bw)		Height (cm) [P]		Weight (kg) [P]		BMI (kg/m^2^) [P]		Waist circumference (cm)		Tanner stage	
	Last visit		Initial	Last visit	Before	Last visit	Before	Last visit	Before	Last visit	Before	Last visit	Before	Last visit
1	24 years	36	0.08	0.13	171 [NA]	171 [NA]	75.4 [NA]	72.3 [NA]	25.8 [NA]	24.7 [NA]	83	80	NA	NA
2	4 years	26	0.013	0.055	89 [95]	107 [95]	12.9 [50]	14.2 [25]	16.3 [50]	12.4 [< 3]	55	49	I	I
3	4 years, 9 months	21	0.015	0.078	103 [>97]	119 [>97]	17.5 [97]	23 [97]	16.5 [55]	16.2 [75]	56	52	I	I
4	25 years	63	0.08	0.1	151 [NA]	151 [NA]	41 [NA]	39 [NA]	17.9 [NA]	17.1 [NA]	61	59	NA	NA
5	12 years	63	0.02	0.06	145 [>97]	170 [>97]	33 [90]	56 [92]	15.7 [25]	19.4 [60]	83	78	I	IV
6	12 years	63	0.02	0.05	146 [>97]	169 [>97]	33.4 [90]	55.6 [92]	15.8 [25]	19.5 [60]	82	78	I	IV
7	10 years, 8 months	9	0.04	0.08	123 [5]	127 [<3]	21.7 [3]	21.8 [<3]	14.3 [<3]	13.5 [<3]	58	50	I	I
8	13 years	69	0.06	0.08	141 [97]	150 [25]	27.1 [55]	29.1 [<3]	13.6 [3]	12.9 [<3]	68	57	I	III
9	44 years	12	0.08	0.24	163 [NA]	163 [NA]	85.7 [NA]	87 [NA]	32.3 [NA]	32.7 [NA]	106	108	NA	NA

*bw* body weight, *P* percentile, *NA* not applicable

Special concerns about weight loss or insufficient weight gain have arisen with regard to children (Table 2). Patient #2 (BS syndrome) had a decreased baseline weight from the 50th percentile to 25th percentile at the last visit (2 years, 2 months of treatment), and patient #8 (APS) had a decreased baseline weight from 55th percentile to the 3rd percentile (after 5 years, 9 months of treatment). In this last patient, the poor auxological outcomes were probably related to the APS. Patient #7 had experienced badly controlled diabetes mellitus since age 4 years; in this patient, her low percentiles were probably related to this poor metabolic control.

Biochemical parameters are shown in Table 3. All of the patients were euthyroid. Patients with BS syndrome (#1 to #7) were analyzed as a separated group.

**Table 3:** Biochemical data for the lipodystrophic patients before and after metreleptin treatment

Patient #	A1c (%)		Glucose (mmol/L)		TG (mmol/L)		HDL-c (mmol/L)		Insulin (mIU/L)		HOMA-IR		Leptin (ng/mL)	
	Before	Last visit	Before	Last visit	Before	Last visit	Before	Last visit	Before	Last visit	Before	Last visit	Before	Last visit
1	10.4	7.6	14.6	8.7	3.8	2.2	0.8	0.8	NA	NA	NA	NA	0.2	25.5
2	4.8	4.6	4.9	4.4	9.72	7.8	0.54	0.85	29.5	1.5	6.5	0.3	0.5	11.5
3	ND	5.0	4.05	4.1	12.98	1.64	0.49	0.64	163	24.3	29.7	4.5	0.5	3.1
4	15.0	7.5	21.1	16.1	28.22	10.4	0.49	0.98	NA	NA	NA	NA	0.1	11.4
5	8.1	5.4	11.2	8.4	25.8	3.43	0.52	0.67	145	42.1	73.2	15.9	0.2	28.9
6	5.9	5.6	5.9	5.8	20.3	1.93	0.65	0.8	181	64.7	48.1	16.9	0.2	49.7
7	13.6	6.3	16.6	7.5	6.45	1.11	0.54	0.59	NA	NA	NA	NA	1.7	26
Mean ± SD	9.6 ± 4.1	6.0 ± 1.2*	11.2 ± 6.6	7.9 ± 4.1	15.3 ± 9.6	3.1 ± 3.3*	0.58 ± 0.1	0.76 ± 0.1*	130 ± 84	33 ± 26*	39 ± 29	9.4 ± 8*	0.5 ± 0.6	22 ± 15*
8	10.6	7.5	9.7	4.4	32.7	0.94	0.65	0.65	190	8.7	83	1.7	0.7	17.9
9	9.6	11.3	12.7	10.3	0.8	1.9	0.8	0.9	NA	NA	NA	NA	14.4	98
Mean ± SD	9.8 ± 3.5	6.8 ± 2.0*	11.2 ± 5.7	7.7 ± 3.8	15.6 ± 11.5	2.7 ± 3.0*	0.61 ± 0.1	0.76 ± 0.1*	141 ± 65	28 ± 26*	48 ± 33	7.9 ± 7*	2.1 ± 4.7	30 ± 29*

Patient #	AST (IU/L)		ALT (IU/L)		GGT (IU/L)		Cr (μmol/L)		UA (μmol/L)	
	Before	Last visit	Before	Last visit	Before	Last visit	Before	Last visit	Before	Last visit
1	34	18	54	46	23	19	53	35	315	309
2	75	33	170	31	43	27	27	35	357	244
3	304	49	158	59	83	34	27	27	226	256
4	140	12	110	25	241	15	ND	62	ND	422
5	100	22	130	58	81	21	ND	44	ND	357
6	83	13	121	33	68	16	ND	44	ND	446
7	17	23	36	22	18	ND	18	20	208	200
Mean ± SD	108 ± 96	24 ± 13*	105 ± 46	39 ± 15*	79 ± 76	22 ± 11*	31 ± 20	38 ± 14	277 ± 156	319 ± 93
8	141	34	302	28	324	99	35	27	ND	202
9	19	22	19	25	32	46	71	53	178	208
Mean ± SD	101 ± 89	25 ± 12*	122 ± 86	36 ± 14*	101 ± 107	35 ± 28*	39 ± 25	39 ± 13	257 ± 146	294 ± 95

*TG* triglyceride, *NA* not applicable because of insulin treatment, *ND* not determined, *AST* aspartate transaminase, *ALT* alanine aminotransferase/alanine transaminase, *GGT* gamma-glutamyltransferase, *Cr* creatinine, *UA* uric acid, *ND* not determined

* *p* < 0.05 vs before metreleptin treatment

Regarding metabolic control, all BS patients with diabetes achieved acceptable Hb A1c values during the first year (from 11.8 to 6.7 % on average), except patient #4. This patient had poor adherence (Fig. 1a). At the last visit, Hb A1c was 2.97 % points lower on average compared with the starting values in this group of patients. On the other hand, the insulin dose could be reduced in patient #1 and #7, from 2.2 and 3.2 IU/kg to 0.6 and 1.89 IU/kg, respectively. Other anti-diabetic medications (metformin and pioglitazone) could be stopped during the treatment, but, except in patient #6, had to be re-introduced later because of worsening HbA1c values.

Insulin sensitivity improved after metreleptin treatment in all patients with the exception of the patient #4, as evaluated by basal insulin plasma levels, HOMA-IR index (Table 3), or insulin requirements.

Metreleptin halved plasma triglycerides levels (Fig. 1b) after 3 months of metreleptin treatment in patients with BS syndrome. At the last visit, plasma triglycerides were reduced by 78 % in this group of patients (Table 3). Except in patient #4, fenofibrate or n-3 free fatty acids were stopped at the beginning of treatment. Also, HDL-c was significantly increased by 31 % (Table 3).

**Fig. 2:**
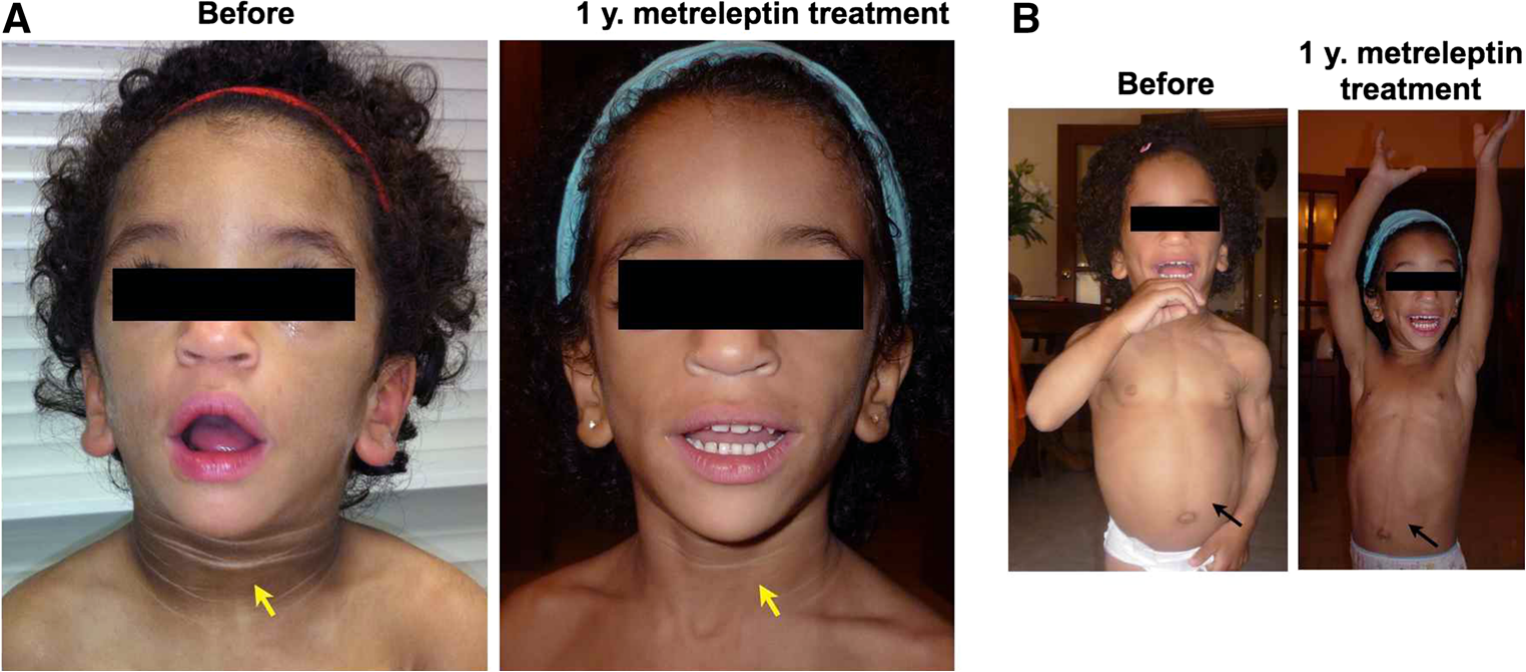
Effect of metreleptin on a young girl (age 23 months old, patient #2) with regard to acanthosis nigricans and hepatic steatosis. *Arrows* show the improvement in the skin lesions (**a**) and the reduction in abdominal circumference (**b**)

All of the BS patients had hepatic steatosis as evaluated by ultrasonography, and except patient #7, they also had nonalcoholic steatohepatitis (NASH) (Table 3). In the first trimester alone, metreleptin reduced both AST and ALT by 30 %; and, at the last visit, this decrease in AST and ALT deepened to 74 and 61 %, respectively (Table 3). As a surrogate endpoint of hepatomegaly, waist circumference was reduced in all patients by an average of 4.6 cm (Table 2; Fig. 2b). In patients #2 and #3, acanthosis nigricans significantly improved (Fig. 2a) after 1 year of treatment. The metreleptin dose was modified according the main endpoints (Hb A1c, triglycerides and transaminases) and weight loss.

The patient with APS (#8) experienced changes in Hb A1c, triglycerides and, plasma transaminases similar to those observed in BS patients. This patient underwent a heart transplant at age 12 years because of a severe dilated cardiomyopathy. Both metabolic control and triglycerides levels worsened after surgery (Fig. 1), probably in relation to severe stress and glucocorticoid treatment.

The patient with FPLD (#9) was the only one in this cohort for whom metreleptin did not improve metabolic control despite a very high dose [0.24 mg/(kg day)]. The treatment was stopped after 1 year in agreement with the patient.

## Discussion

In this study, we confirm that metreleptin significantly improved metabolic and hepatic complications in patients with genetic generalized lipodystrophy, and its effects were maintained for long periods of time (more than 5 years in four patients). No relevant side effects were reported, and the weight loss was in the range of other studies [9].

Metreleptin treatment was not effective, however, in improving metabolic control in the only patient with type 2 FPLD; although the drug allowed cessation of lipid-lowering medication, this patient’s triglycerides levels were lower with the standard medication (fenofibrate plus n-3 free fatty acid). A previous group [4] reported that metreleptin was effective for decreasing Hb A1c in five of six patients with type 2 FPLD over 12 months of treatment and was effective in all of them for decreasing triglycerides. However, the majority of these patients had low baseline leptin concentrations (<5 ng/mL), while our patient had higher baseline leptin levels (14.4 ng/mL). Because the degree of hypoleptinemia seems to be critical in the effectiveness of metreleptin treatment [10], Simha et al. [11] compared the effect of this treatment in two groups of patients with type 2 FPLD, one with severe hypoleptinemia (SH, 1.9 ng/mL on average) and the other with moderate hypoleptinemia (MH, 5.3 ng/mL on average). They concluded that metreleptin replacement therapy was equally effective in FPLD patients with both SH and MH in reducing serum and hepatic triglyceride levels but did not improve hyperglycaemia. In a more extended study of metreleptin treatment, Chan et al. [5] enrolled 14 people with FPLD, and although the global results on metabolic control, lipid profile, and hepatic steatosis were good, no specific information about patients with FPLD was provided. Taken together, the evidence suggests that severe hypoleptinemia could be a determinant of the magnitude of improvement of metabolic control in patients with FPLD who are treated with metreleptin.

Focusing on BS patients, metreleptin reduced Hb A1c by 2.97 points in agreement with previous reports [5]. Also, the reduction of triglycerides was remarkable (78 %). Chan et al. [5] reported a similar reduction (73 %) after three years of treatment. Strikingly, HDL-c levels significantly increased (31 %), whereas other studies found no changes in HDL-c [4, 5, 9, 11], although a tendency to increase was observed in the US National Institutes of Health study [5]. We do not have a clear explanation for this discrepancy, but a longer period with low triglycerides levels might be one possibility.

Insulin sensitivity improved in all patients with generalized lipodystrophy except in patient #4, as measured by HOMA, plasma insulin level reduction, or lower insulin requirement. In those patients without insulin treatment, the basal insulin level reduction ranged from 64 to 95 %. The improvement in insulin sensitivity after metreleptin has been reported by others using different approaches [9, 12–14]. The mechanisms responsible for insulin resistance reduction observed during metreleptin treatment continue to be a matter of controversy and are beyond the current scope; however, the reduction in lipid accumulation in both liver and muscles—along with the resulting lower lipid toxicity probably associated with a lower energy uptake—seems to be a plausible explanation [6].

The plasma insulin reduction would explain the significant improvement in acanthosis nigricans observed in the two younger children; however, this change did not occur in the older patients despite improved in insulin sensitivity. This result underlines the importance of starting metreleptin replacement as soon as possible.

Hepatic steatosis and NASH are common complications of these rare lipodystrophic syndromes, which in some cases can evolve to cirrhosis. All patients had hepatic steatosis as evaluated by liver ultrasonography, and seven also had NASH. In less than 6 months, we observed a significant reduction in liver enzymes after metreleptin treatment, which was sustained over time, and also a reduction in abdominal circumference (Table 2). Others have also reported improvement in hepatic enzymes, as a surrogate marker of NASH, after metreleptin treatment [5, 12, 13, 15]. Recently, Safar Zadeh et al. [16], analyzing hepatic biopsies, demonstrated that leptin replacement reversed hepatic steatosis and NASH to a significant degree. Although they were unable to identify an improvement in fibrosis, their patients showed no progression of this damage. The precise mechanism of leptin action on fatty liver is still poorly understood. Leptin acts at the hypothalamus, reducing appetite, so a decrease in energy uptake would potentially allow for mobilization of stored triglycerides from the liver [14, 15].

Six of the nine studied patients were children under age 9 years (age range 23 months to 8.8 years of age). In all six, metreleptin was effective in terms of metabolic control, triglyceride reduction, and fatty liver disease improvement, for more than 21 months on metreleptin except patient #7 (9 months), and more than 5 years in four patients. These results contrast with those reported by Beltrand et al. [17], who identified partial or total resistance after 28 months of metreleptin replacement in five of eight children with BS syndrome. The authors argued that a possible cause of this resistance was the presence of neutralizing anti-leptin antibodies, measured in two patients. This factor as a cause of reduced effectiveness in lipodystrophic patients on metreleptin has not been reported elsewhere, but has been reported in patients with congenital leptin deficiency under similar treatment [18]. On the other hand, in the largest studied cohort [5], with a 53 % pediatric population, no mention was made of an effect reduction of or resistance to metreleptin treatment over at least three years of treatment. All of these data reinforce the need for more extended studies in pediatric populations with generalized lipodystrophy to establish the real effectiveness of this treatment.

To the best of our knowledge, patient #8 is the first case reported with APS to be treated with metreleptin for more than 5 years. At the age of 8 years, this patient was diagnosed with diabetes mellitus, severe hypertriglyceridemia, NASH, and dilated cardiomyopathy, and started treatment with metreleptin. Metreleptin was successful in controlling the metabolic and hepatic complications; however, his heart disease worsened, and at age of 12, the patient entered the final stages of his cardiac function with a very limited quality of life. Because of his perfect metabolic control and normal transaminase levels, we decided, in agreement with the patient and his parents, to submit the case to our regional pediatric transplant commission and the boy underwent a successful heart transplant in May 2013. After surgery, the patient suffered a worsening of glucose metabolism and lipid profile, probably because of glucocorticoid treatment; however, after increasing metreleptin dose and the addition of metformin, these biochemical parameters improved significantly.

In summary, with this study, we extend the experience with the effectiveness of metreleptin in the treatment of genetic lipodystrophies. This hormone is effective for long periods in people with generalized lipodystrophy associated with severe hypoleptinemia for controlling diabetes, hypertriglyceridemia, and hepatic steatosis, without remarkable side effects.
